# Crop Safety and Weed Control of Foliar Application of Penoxsulam in Foxtail Millet

**DOI:** 10.3390/plants13162296

**Published:** 2024-08-18

**Authors:** Shuqi Dong, Tingting Chen, Ruize Xi, Shulin Gao, Gaofeng Li, Xuena Zhou, Xie Song, Yongqing Ma, Chunyan Hu, Xiangyang Yuan

**Affiliations:** 1 College of Agriculture, Shanxi Agricultural University, Jinzhong 030800, China; dongshuqi@sxau.edu.cn (S.D.); sxndctt@163.com (T.C.); x1203618792@163.com (R.X.); gaoshulin1005@163.com (S.G.); ligaofeng6718@163.com (G.L.); sxndsxe@163.com (X.S.); 2 College of Plant Protection, Shanxi Agricultural University, Jinzhong 030800, China; zxn13546233734@163.com; 3 Institute of Soil and Water Conservation, Chinese Academy of Sciences (CAS) & Ministry of Water Resources (MWR), Xianyang 712100, China; mayongqing@ms.iswc.ac.cn

**Keywords:** foxtail millet, penoxsulam, antioxidant system, weed control

## Abstract

Grass damage has become an important factor restricting foxtail millet production; chemical weeding can help resolve this issue. However, special herbicides in foxtail millet fields are lacking. Penoxsulam has a broad weed control spectrum and a good control effect. In this project, Jingu 21 was used as the test material, and five different concentrations of penoxsulam were used for spraying test in the three–five leaf stage. In this experiment, the effects on the growth of foxtail millet were discussed by measuring the agronomic characters and antioxidant capacity of foxtail millet after spraying penoxsulam. The results showed that: (1) penoxsulam is particularly effective in controlling *Amaranthus retroflexus* L. *(A. retroflexus)* and *Echinochloa crus-galli* (L.) Beauv. (*E. crus-galli)*, but is ineffective in controlling *Chenopodium album* L. (*C. album*) and *Digitaria sanguinalis* (L.) Scop. (*D. sanguinalis*); (2) the stem diameter, fresh weight, and dry weight of the above-ground parts decreased with the increase in spraying amount; (3) as the spraying dosage increased, the superoxide (SOD), peroxidase (POD), and catalase (CAT) activities in the foxtail millet initially increased and subsequently decreased; the malonaldehyde (MDA) content increased. Our experiment found that 1/2X and 1X spraying dosages had certain application value in controlling gramineous weeds in foxtail millet field. Other spraying dosages are not recommended as they may harm the crops. Our findings provide reference for identifying new herbicides in the foxtail millet field.

## 1. Introduction

Foxtail millet is one of the world’s oldest domesticated cereals. It was first domesticated 11,000 years ago, and became the dominant crop in dry farming ecological agriculture [1,2]. Foxtail millet has numerous advantages, including drought resistance, tolerance to poor soil, disease and pest resistance, wide adaptability, and nutrient richness. Foxtail millet has been widely planted in arid and semi-arid areas [3,4,5].

There is competition for water, fertilizer, air, and light between weeds and crops, which seriously affects the growth and development of crops and inevitably also affects the yield and quality [6,7,8]. Weed control is an important problem in agricultural production [9]. Chemical weeding has played an indispensable role in the process of agricultural modernization globally [10]. Despite reducing the damage caused by weeds, herbicides may make weeds resistant to herbicides [11].

Penoxsulam is a herbicide mainly composed of triazolopyrimidine sulfonamide active ingredients, and its chemical formula is C_16_H_14_F_5_N_5_O_5_S [12]. It can inhibit the germination of weed seeds and the growth of tender tissues, has a strong control effect on gramineous weeds, and can be used in most areas in China. It spreads through the plant roots, leaves, and buds [13]. It has been applied internationally to prevent broad-leaved weeds in soybean, cotton, and sugar beet fields. Penoxsulam is a water-soluble herbicide characterized by good water solubility and non-volatility. Compared with other herbicides, it is slowly hydrolyzed in soil and absorbed by plants. This product exhibits a high weeding effect and good crop protection. Because of its unique mechanism of action and excellent selectivity, it is a widely used herbicide for preventing gramineous weeds and broadleaf grasses. Penoxsulam has shown excellent control effects on annual and perennial weeds, particularly broadleaf weeds [14]. It has been widely used in the control of various Gramineae and Leguminosae weed species in other countries. This is an innovative herbicide, whose main mechanism of action is to control weeds by inhibiting the activity of acetolactate synthase (ALS), thus limiting the biosynthesis process of the branched-chain amino acids, leucine, isoleucine, and valine [15,16].

Crops are often stressed by single or multiple herbicides. The stress of exogenous herbicides can induce cells to produce reactive oxygen species (ROS), which in turn leads to membrane lipid peroxidation, protein denaturation, and other damage. Plant antioxidant enzymes such as superoxide (SOD), peroxidase (POD), and catalase (CAT) are all affected by herbicide stress [17]. Akbulut et al. [18] found that atrazine inhibited plant peroxidase, ascorbic acid peroxidase, and lipid peroxidation during the 15-day experiment. The accumulation of active oxygen (ROS) in plants under stress (herbicide) can cause lipid peroxidation and MDA production [19]. The same phenomenon is observed in numerous herbicide-treated plants [20,21].

Although the application of herbicides on crops is an effective weeding measure, herbicides can also adversely affect growing crops, including injury, delayed growth, and reduced yield [22]. Dash et al. [23] showed that spraying 25 g/hm^2^ penoxsulam after 20 days of rice transplanting could effectively manage the compound weed flora and increase the yield of summer rice. Yadav et al. [24] showed that spraying 22.5 g/hm^2^ penoxsulam could reduce the weed density (0.0–0.7 g/m^2^) and dry weight (0.0–6.3 g/m^2^) in transplanted rice fields. As a common pre-emergence herbicide for weeds in rice fields, butachlor showed a good yield-increasing effect in production [25,26]. Studies on penoxsulam have primarily focused on its influence on rice safety and rice control [12,27], investigation of resistant genes [28,29], effect of compounding with other herbicides [30], and the residual dissolution of penoxsulam in rice and soil [31,32]. However, reports related to penoxsulam in foxtail millet fields are few. Therefore, the aim of this study was to investigate the effects of different doses of penoxsulam on (i) growth, (ii) antioxidant enzyme activities and contents, and (iii) yield traits and yield of foxtail millet, to provide reference for identifying suitable herbicides in foxtail millet fields.

## 2. Results

### 2.1. Effects on Weed Control by Spraying Penoxsulam

**Figure 1:**
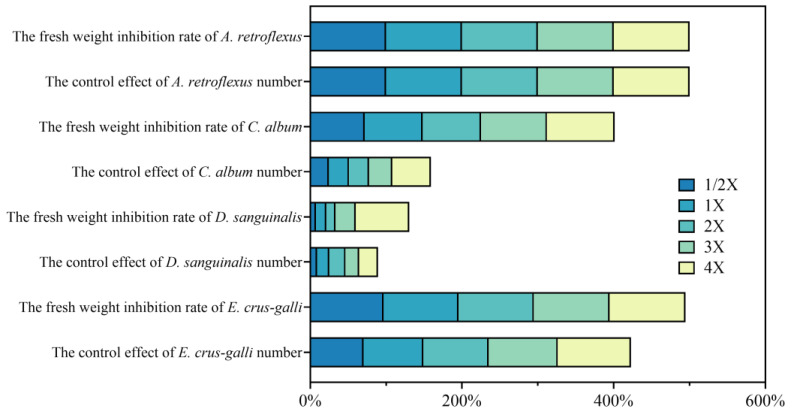
Effects of penoxsulam on weed control.

As shown in Figure 1, penoxsulam has some effect on *A. retroflexus*, *C. album*, *D. sanguinalis*, and *E. crus-galli*, and has the best control effect on *A. retroflexus* and *E. crus-galli*. The prevention and control effect of penoxsulam increases with increasing the spraying time. The effect of penoxsulam on *A. retroflexus* and *E. crus-galli* improved gradually with the increase in spraying time. The control effect of *E. crus-galli* was higher than 70% at all treatments after 20 days, and the fresh weight inhibition rate of *E. crus-galli* was higher than 95% at 20 days after spraying. There was no significant difference in the control effect of *D. sanguinalis* under different spraying doses. The control effect of *C. album* was less than 55% at 20 days after application and less than 30% at all spraying dosages except 3X and 4X. The fresh weight inhibition rate of *C. album* exceeded 65% at all spraying dosages. After 20 days, the control effect of *A. retroflexus* was 100%. The inhibition rate of fresh weight of *A. retroflexus* was above 92% at 20 days after application (Figure 1).

### 2.2. Effects on Agronomic Traits by Spraying Penoxsulam

#### 2.2.1. Effects on Stem Diameter of Foxtail Millet

**Figure 2:**
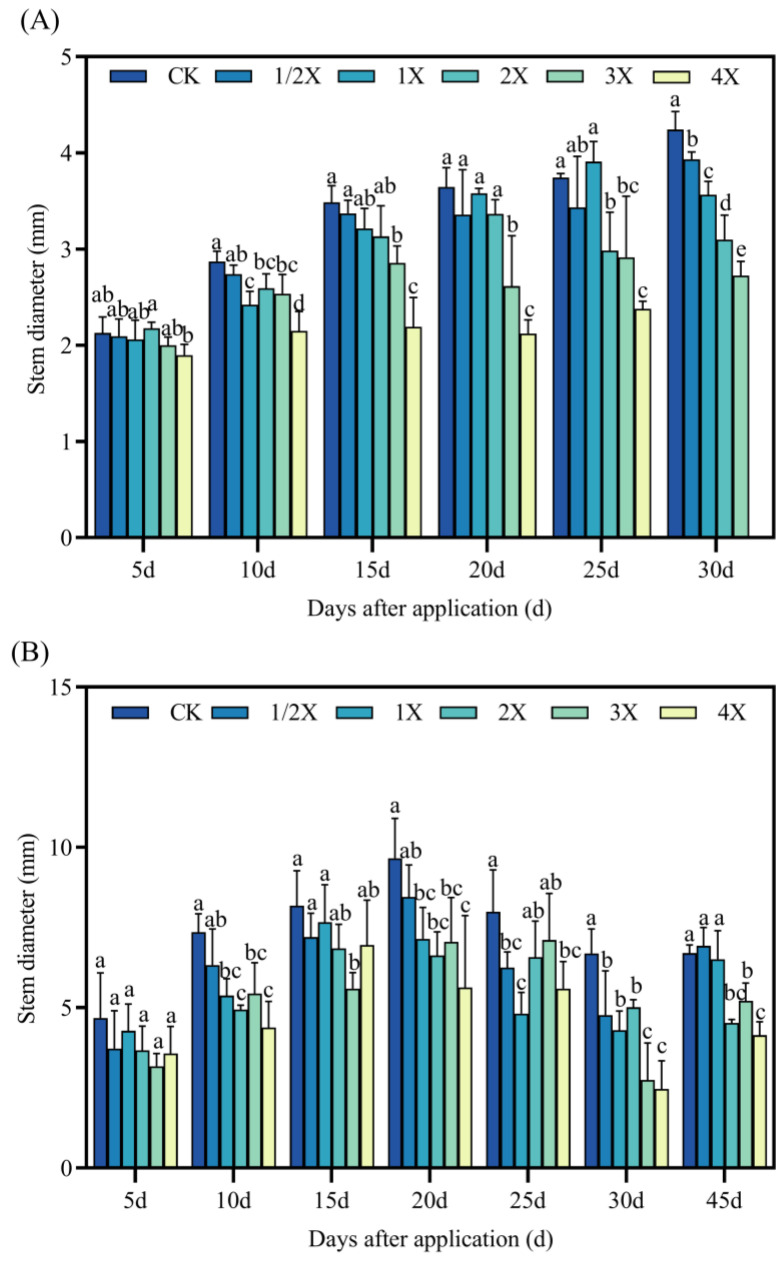
Effect on stem diameter. (**A**) Pot plant experiment; (**B**) field experiment. After 30 days of spraying in pot experiment, all the foxtail millet treated with 4X died. Significant differences between treatments with different concentrations are indicated by lowercase letters (*p* < 0.05).

As seen in Figure 2, whether in the pot or field experiments, the stem diameter of Jingu 21 decreases to a certain extent after penoxsulam treatment, and the stem diameter gradually decreases with increasing the spraying dose.

The stem diameter of Jingu 21 was higher than that of CK at the 5th day in the pot experiments. However, the difference was not significant. At 15 days after spraying, the stem diameter decreases by 3–37% compared with CK under different spraying dosages. Then, 5–25 days after spraying, 1/2X lower stem diameter than CK, but no significant difference was found. Compared with CK, the stem diameter of the 4X spraying dosage decreases by 44% at 20 days after treatment. In the field, the stem diameter of all spraying doses and CK was not significantly different at 5 days after application. At 15 days after spraying, the stem diameter under 3X spraying dosage decreases by 32% compared with CK. After 25 days, the stem diameter of 1X spraying was significantly reduced by 40% than that of CK. After 30 days, the stem diameter increases by 29–63% compared with CK. At 45 days after treatment, the stem diameter of the 1/2X spraying dosage increases by 3% compared with CK (Figure 2).

#### 2.2.2. Effects on Above-Ground Fresh Weight of Foxtail Millet

**Figure 3:**
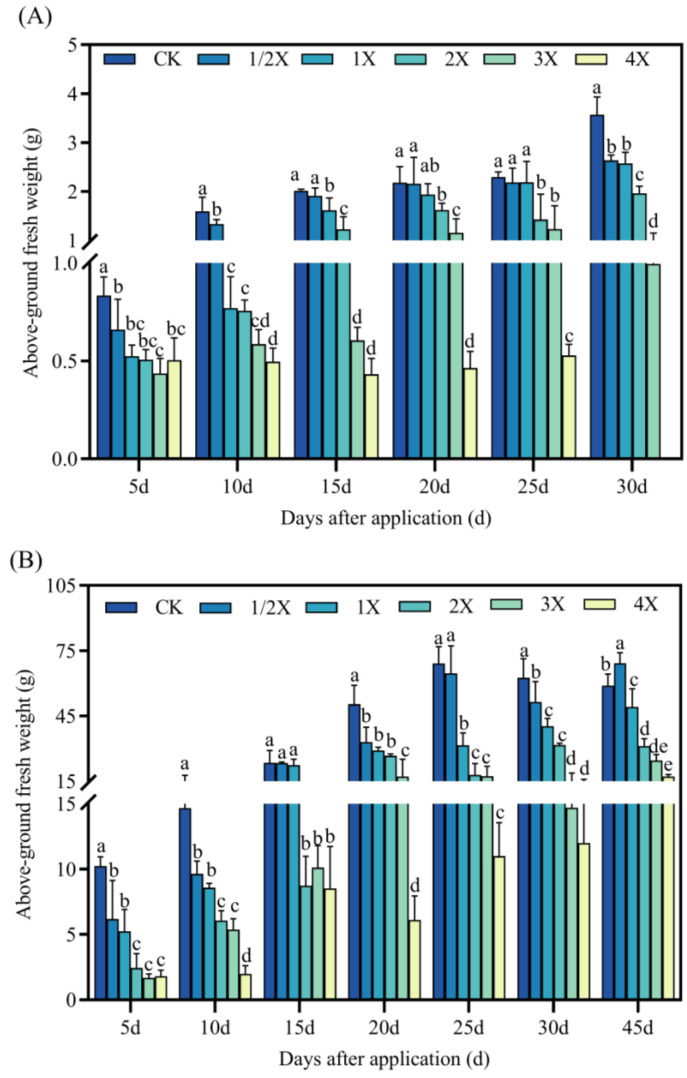
Effect on above-ground fresh weight. (**A**) Pot plant experiment; (**B**) field experiment. After 30 days of spraying in pot experiment, all the foxtail millet treated with 4X died. Significant differences between treatments with different concentrations are indicated by lowercase letters (*p* < 0.05).

As seen in Figure 3, in the pot and field experiments, the above-ground fresh weight of Jingu 21 decreases to a certain extent after penoxsulam application, and the general trend is that the above-ground fresh weight decreases as the amount of spraying increases.

In the pot experiment, the above-ground fresh weight decreases by 48% in comparison with CK at 5 days after spraying with 3X the spraying dosage. After 10 days, the fresh weight of above ground under different spraying dosages decreases by 14–69% compared with CK. Then, 15 days after treatment, the above-ground fresh weight under the 4X spraying dosage decreases by 79% compared with CK. There was no significant difference between the above-ground fresh weight and CK under the spraying dosages of 1/2X and 1X at 20–25 days after treatment. In the field experiment, the above-ground fresh weight was significantly reduced by 79% compared with CK at 5 days under the 2X spraying dosage. After 20 days, the above-ground fresh weight was significantly reduced by 84% under the 3X spraying dosage compared with CK. After 30 days, the above-ground fresh weight under different spraying dosages decreases by 18–92% compared with CK. After 45 days, the above-ground fresh weight increases by 15% compared with CK at the 1/2X spraying dosage (Figure 3).

#### 2.2.3. Effects on Above-Ground Dry Weight of Foxtail Millet

**Figure 4:**
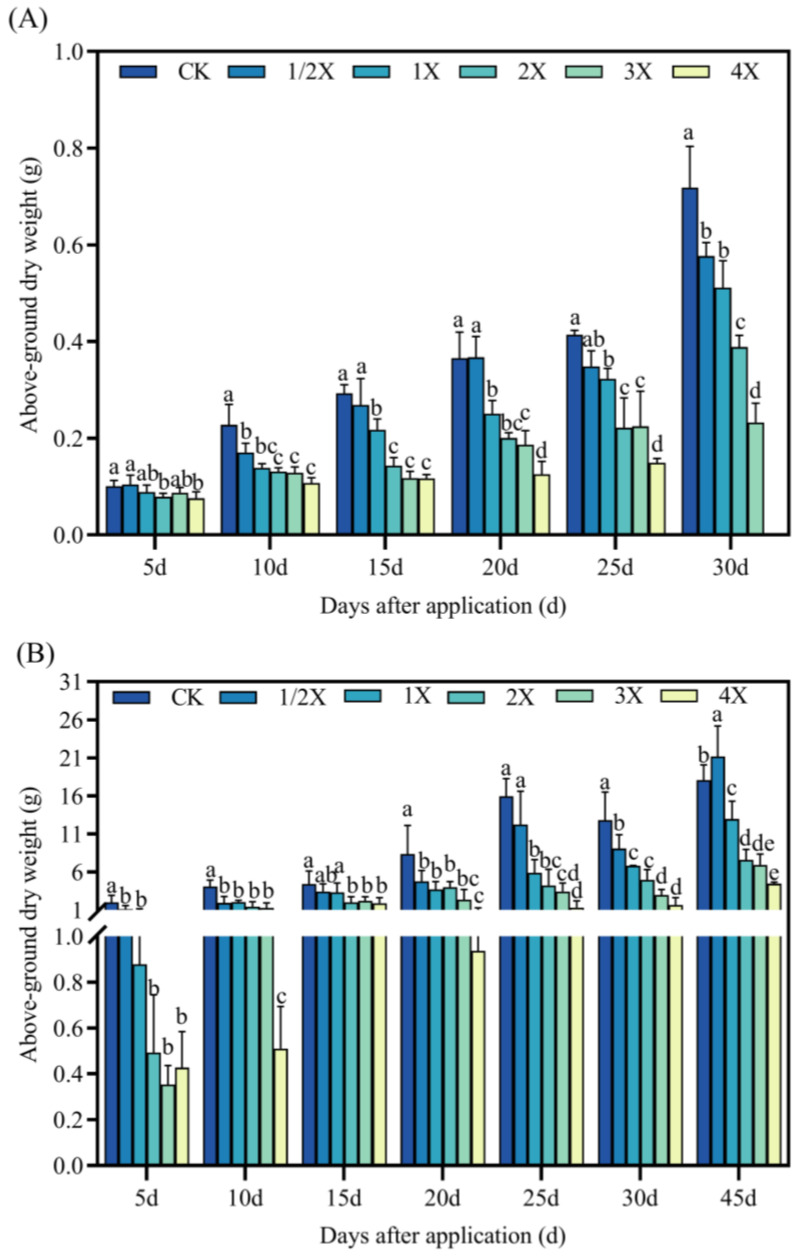
Effect on above-ground dry weight. (**A**) Pot plant experiment; (**B**) field experiment. After 30 days of spraying in pot experiment, all the foxtail millet treated with 4X died. Significant differences between treatments with different concentrations are indicated by lowercase letters (*p* < 0.05).

As seen in Figure 4, in both the pot and field experiments, penoxsulam treatment causes a decrease in the above-ground dry weight. The overall trend showed that as the spraying dosage increases, the dry weight of the above-ground parts gradually decreases.

In the pot experiment, the above-ground dry weight decreases by 24% compared to CK after 5 days at a spraying dosage of 4X. Fifteen days after spraying, the above-ground dry weight at a spraying dosage of 3X decreases by 60% compared to CK. After 20 days, the above-ground dry weigh under the 4X spraying dosage decreases by 66% compared with CK. Compared with CK, the above-ground dry weight decreases by 20–68% 30 days after treatment. In the field experiment, the above-ground dry weight decreases by 79% in comparison with CK in 5 days under the 3X dosage. At 10 days after spraying, the above-ground dry weight was significantly reduced by 92% under the 4X spraying dosage compared with CK. At 15 days, the above-ground dry weight under the 2X spraying dosage decreases by 55% compared with CK. Compared with CK, the above-ground dry weight of the shoot at 1X, 2X, 3X, and 4X spraying dosages decreases by 52–88% at 20 days after treatment. At 30 days after treatment, the above-ground dry weight was significantly reduced by 86% under the 4X spraying dosage compared with CK. At 45 days after treatment, the above-ground dry weight increases by 25% compared with CK at the 1/2X spraying dosage (Figure 4).

### 2.3. Effects on SOD, POD, CAT, Activities and MDA Content of Foxtail Millet

**Figure 5:**
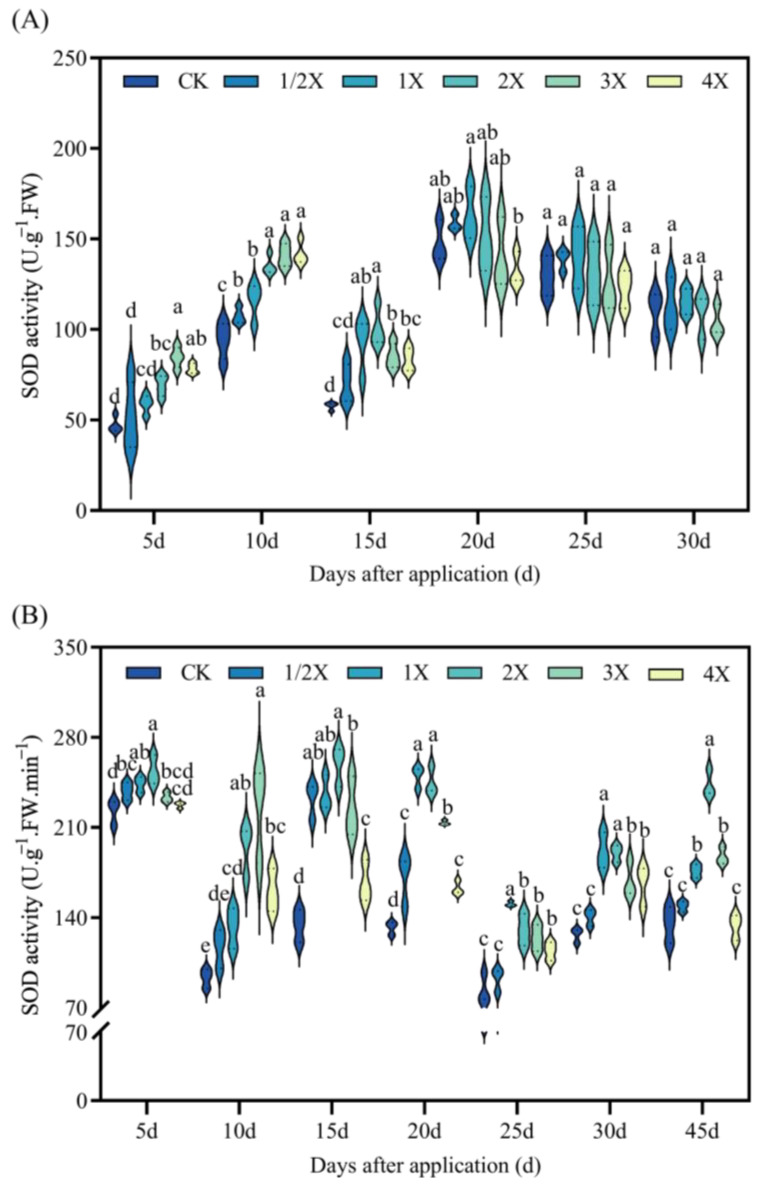
Effect on SOD activity. (**A**) Pot plant experiment; (**B**) field experiment. After 30 days of spraying in pot experiment, all the foxtail millet treated with 4X died. Significant differences between treatments with different concentrations are indicated by lowercase letters (*p* < 0.05).

In Figure 5, the SOD activity of foxtail millet is shown as initially increasing, and subsequently decreasing with the increasing spraying dose.

Compared with CK, the SOD activity of 5 days under 3X increases by 80% in the pot experiment. At 15 days, the SOD activity increases by 74% under the 2X spraying dosage compared with CK. After 20–30 days, there was no significant difference in SOD activity between the different spraying dosages and CK. In the field experiment, SOD activity significantly increases by 108% and 142% compared with CK after 10 days, under the 2X and 3X spraying dosages. At 15 days after treatment, SOD activity increases by 93% compared with CK under 2X treatment. At 25 days, SOD activity increases by 80% compared with CK under 1X treatment. At 45 days, the activity of SOD increases 80% than that of CK under 2X treatment (Figure 5).

**Figure 6:**
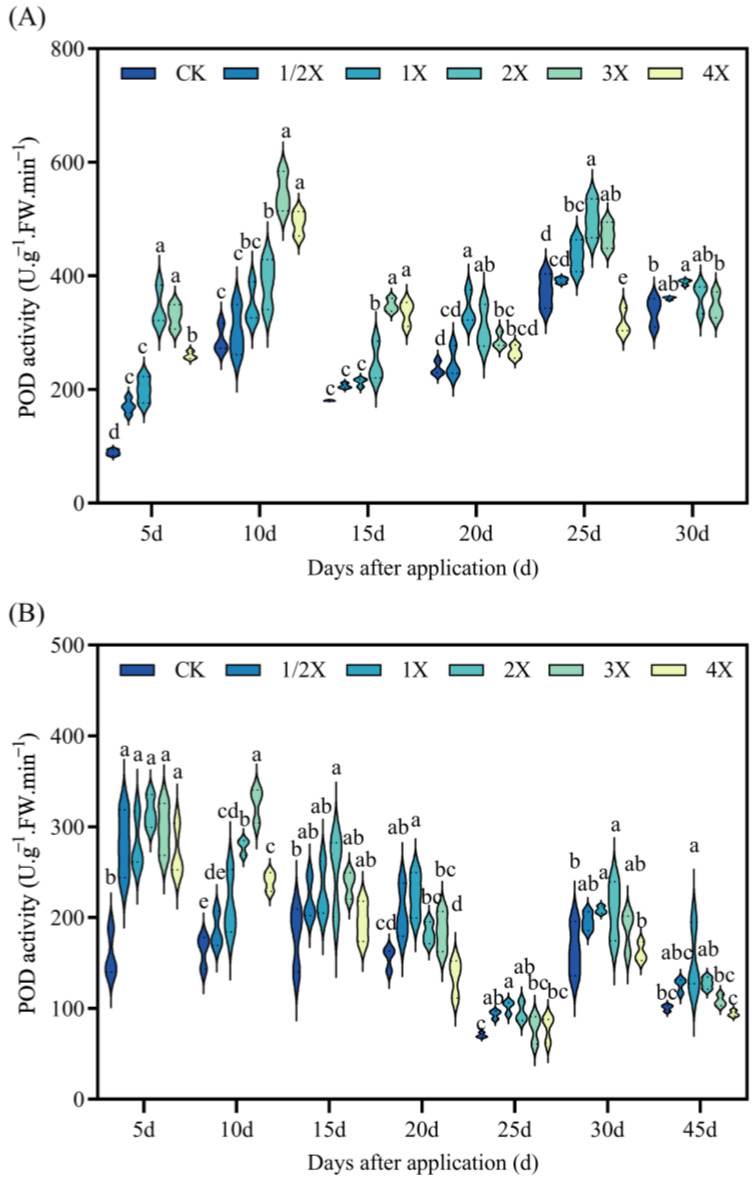
Effect on POD activity. (**A**) Pot plant experiment; (**B**) field experiment. After 30 days of spraying in pot experiment, all the foxtail millet treated with 4X died. Significant differences between treatments with different concentrations are indicated by lowercase letters (*p* < 0.05).

As seen in Figure 6, in the pot and field experiments, the activity of POD increases at first, and then decreases as the amount of spraying increases.

In the pot experiment, the POD activity significantly increases by 285% and 270% compared with CK after 5 days, under the 2X and 3X treatments. After 15 days, POD activity increases by 31% under the 3X spraying dosage compared with CK. After 20 days, POD activity increases by 44% compared with CK under the 1X spraying dosage. Subsequently, 25 days after treatment, POD activity decreases by 15% under the 4X spraying dosage compared with CK. The activity of POD was not significantly different from that of CK at 30 d. In the field experiment, the POD activity of Jingu 21 significantly increases by 100% compared with CK at 5 days under 2X spraying dosage. At 10 days after spraying, the POD activity increases by 99% under the 3X spraying dosage compared with CK. After 15 days, the POD activity increases by 40% compared with CK under the 2X spraying dosage. After 20 days, the POD activity increases by 45% compared with CK under the 1X spraying dosage. After 45 days, the POD activity increases by 49% compared with CK at 1X spraying dosage (Figure 6).

**Figure 7:**
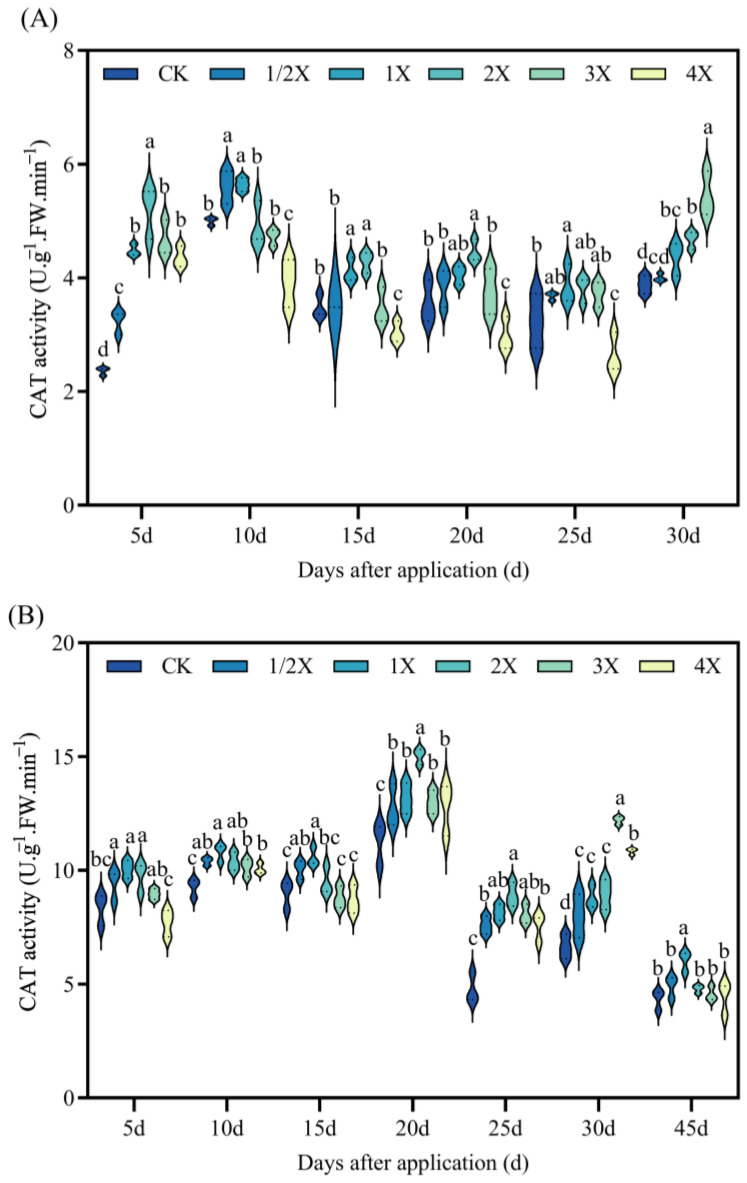
Effect on CAT activity. (**A**) Pot plant experiment; (**B**) field experiment. After 30 days of spraying in pot experiment, all the foxtail millet treated with 4X died. Significant differences between treatments with different concentrations are indicated by lowercase letters (*p* < 0.05).

As seen in Figure 7, in both the pot experiment and field experiment, after penoxsulam treatment, the CAT activity initially increases and subsequently decreases with the increase in the amount of penoxsulam.

In the pot experiment, the CAT activity increases by 120% compared with CK, 5 days after spraying with the 2X spraying dosage. At 10 days after treatment, CAT activity significantly decreases by 22% compared with CK under the 4X treatment. After 20 days, the CAT activity increases by 25% compared with CK under the 2X spraying dosage. The CAT activity increases by 12–41% compared with that of CK at 30 days, under the 1X, 2X, and 3X spraying dosage. In the field experiment, the CAT activity significantly increases by 20% compared with CK at 5 days under the 1X spraying dosage. After 20 days, the CAT activity increases by 13–33% compared with CK. At 25 days, the CAT activity increases by 87% compared with CK under the 2X spraying dosage. After 30 days, the CAT activity increases by 84% compared with that of CK at 3X spraying dosage. At 45 days after treatment, the CAT activity increases by 39% compared with CK at the 1X spraying dosage (Figure 7).

**Figure 8:**
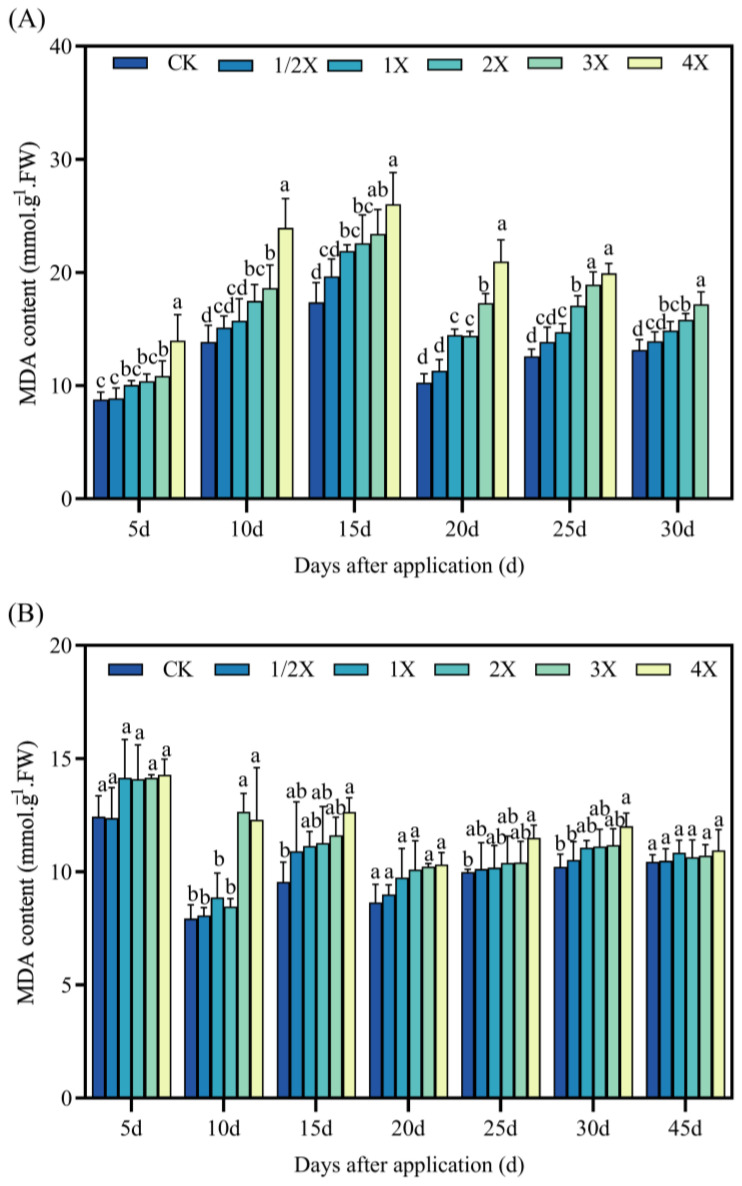
Effect on MDA content. (**A**) Pot plant experiment; (**B**) field experiment. After 30 days of spraying in pot experiment, all the foxtail millet treated with 4X died. Significant differences between treatments with different concentrations are indicated by lowercase letters (*p* < 0.05).

Figure 8 shows that in both experiments, the MDA content shows an increasing trend after being treated with penoxsulam.

Under the pot experiment, the MDA content significantly increases by 58% compared with CK after spraying for 5 days with the 4X spraying dosage. Compared with CK, the MDA content increases by 9% and 19% under the 3X and 4X treatments at 10 days. At 15 days, the MDA content increases by 30–62% compared with CK. At 20 days after treatment, the MDA content under the 4X spraying dosage increases by 68% compared with CK. After 30 days, the MDA content of CK was not significantly different from that of all treatments. In the field experiment, no significant difference is observed between the MDA content of all spraying dosages and CK at 5 days. At 10 days after spraying, the MDA content increases by 59% compared with that of CK under the 3X spraying dosage. At 20 days, there was no significant difference between the MDA content of the CK and all dosages. After 30 days, the MDA content of the 4X spraying increases by 17% compared with CK. At 45 days, no significant difference is found between CK and all spraying dosages (Figure 8).

### 2.4. Effects on Yield Traits of Foxtail Millet by Spraying Penoxsulam

Table 1 shows that under different treatments of penoxsulam, all four indexes of foxtail millet yield traits (ear length, ear weight, 1000 grain weight and yield) show a decreasing trend with the increase in spraying dosage. With the increase in spray dosage, the spike number and yield first increases and then decreases.

Compared with CK, the ear length is significantly reduced by 10% and 17% under the 2X and 4X spraying dosages; the ear weight of the 4X spraying dosage decreases significantly by 30%; the 1000 grain weight of 4X spraying dosage decreases significantly by 12%; and the yield of the 1/2X spraying dosage increases significantly by 24%. Moreover, the yield of the 2X and 3X spraying dosages decrease significantly. However, the difference was not significant, and the yield of the 4X spraying dosage decreases significantly by 23% compared with CK (Table 1).

## 3. Discussion

This experiment found that penoxsulam has good control effects on four weeds. In field experiments, the control effect on *A. retroflexus* and *E. crus-galli* was more optimized than that of *C. album* and *D. sanguinalis*. When the dosage of penoxsulam increases, the control effect also increases. When the concentration of spraying is 4X, its efficacy is maximal. However, penoxsulam has obvious phytotoxicity to foxtail millet after being treated with this dosage.

Plants will change the growth morphology and growth rate of plants to adapt to the stress environment, and the changes in agronomic characters such as plant height, stem diameter, leaf area, and biomass can more intuitively reflect the influence of plants on stress. Herbicides can inhibit the development process of foxtail millet, resulting in a series of effects such as relatively short plants, relatively small leaf area, and relatively low dry matter content. The fomesafen significantly inhibited the height and biomass of seedlings [33]. Weerasooriya et al. [34] concluded that sulfuron-methyl inhibited the growth of sulfonylurea susceptible and resistant plants at 22.5 g/hm^2^, and imasulfuron-methyl inhibited the growth of susceptible plants at 22.5 g/hm^2^. Boulahia et al. [35] showed that 30 days after treatment with prometryne, it had an obvious inhibitory effect on the growth of kidney beans, and had obvious inhibitory effects on bud height, fresh weight, dry weight, and leaf area, so it showed the state of induced pressure. In this experiment, with increasing the spraying dosage of penoxsulam, the inhibitory effect on stem diameter and fresh dry weight of the shoot of foxtail millet gradually increased, which was basically consistent with the previous research results. Whether in pot or field experiments, spraying penoxsulam resulted in an almost significant reduction in the above-ground dry and fresh weight of foxtail millet, and did not gradually decrease after a long period of spraying, which may be related to the inhibition of foxtail millet growth by penoxsulam.

Oxidative stress is a rapid response of plants to adversity. Oxidative stress refers to the imbalance between ROS production and detoxification of active intermediates. There are numerous protective enzymes in plants, such as SOD, POD, and CAT [36]. Many research studies have shown that herbicides can induce lipid peroxidation in leaf cells. Thus, the MDA content can be significantly increased [37,38]. Zhang et al. [39] showed that SiCSD transgenic tobacco plants improved their tolerance to drought, low temperature, and oxidative stress, and showed higher SOD and CAT activities. MDA could inhibit the activity of cytoprotective enzymes and reduce the content of antioxidants, thus aggravating the lipid peroxidation of plant cell membranes [40]. The results of this experiment are basically consistent with the previous research results. After penoxsulam treatment, the activities of SOD, POD, and CAT in the leaves of two kinds of foxtail millet initially increased and then decreased with increasing the spraying dosage, while the MDA content increased with increasing the spraying dosage of penoxsulam. This shows that a relatively low spraying dose of penoxsulam promotes SOD, POD, and CAT activities, and a relatively high spraying dose exceeds the self-regulation range of plants, resulting in SOD, POD, and CAT activities being lower than those under a low spraying dose. However, with prolonged spraying time, the differences between SOD, POD, and CAT activities and MDA contents gradually decrease, indicating that plants can protect their normal growth and metabolism by strengthening the antioxidant enzyme system. Simultaneously, as plants gradually recover, the SOD, POD, and CAT activities in plants are gradually reduced.

Yield composition is an important index of herbicide safety research, and most intuitively reflects whether the herbicide can be used in practical field production. There is a close relationship between grain weight and yield, which are the macro data of comprehensive growth and physiology [41]. The yield of foxtail millet treated with a high dose of tribenuron-methyl was seriously reduced [42]. After spraying penoxsulam, all five yield indexes of foxtail millet (ear length, ear diameter, ear weight, ear grain weight, and 1000 grain weight) showed a decreasing trend with an increasing spraying dose. Yield increased initially and then decreased with increasing the penoxsulam spraying dose, probably because after spraying penoxsulam, the foxtail millet plants were deformed owing to phytotoxicity. The plant deformities under the treatments of the 1/2X and 1X spraying dosages will gradually ease with the extension of days after spraying until they return to normal growth, and at the same time, the foxtail millet will produce tillers to fight phytotoxicity. With the increase in spraying dosage, the time required for the foxtail millet to recover from deformity is prolonged, and the plant cannot recover under the spraying dosage of 4X. However, its tillers increase with the increasing spraying dosage, resulting in a number of effective panicles.

In this experiment, only certain enzymes and active substances in the antioxidant system were studied, and the research was not comprehensive enough. In future, the response of other antioxidant indexes of foxtail millet to penoxsulam need to be studied further. At the same time, considering the poor control effect of penoxsulam on *D. sanguinalis* and *C. album*, we need to further study the combination of penoxsulam and other herbicides, to minimize the harm caused by weeds in fields.

## 4. Materials and Methods

### 4.1. Materials

Object: Jingu 21 (an excellent conventional variety with extreme sensitivity to herbicide, which was selected by Institute of Economic Crops, Shanxi Academy of Agricultural Sciences, Taiyuan, China). Herbicide: 2.5% penoxsulam dispersible oil suspension concentrate (active ingredient: 25 g/L, Dow Yinong Agricultural Science and Technology Co., Ltd. (Nantong, China). Date of production: 6 January 2020, Batch No: 202001060120200307G.

### 4.2. Field Foxtail Millet Test

This experiment was carried out at the Agricultural College of Shanxi Agricultural University. A random and complete block design, with three repeated plots, was adopted in this experiment. At 3–5-leaf stage of foxtail millet, treatment with water (control, CK), 15 (1/2X), 30 (1X), 60 (2X), 90 (3X), and 120 (4X) g a.i. Ha^−1^ penoxsulam was performed. The application was carried out through a laboratory kettle spray, equipped with a nozzle calibration to provide 450 L/ha. The stem diameter, above-ground fresh weight, and dry weight of foxtail millet were recorded at 5, 10, 15, 20, 25, 30, and 45 days after treatment, and its antioxidant enzyme activity and content were measured using sampling methods (Table 2).

### 4.3. Pot Foxtail Millet Test

The pot plant experiment was conducted at the greenhouse, where the durations of light and darkness were 16 h and 8 h, respectively, and the temperature was 25 °C in bright light and 18 °C in the dark. The intensity of light reached 12,000 xl, while the relative humidity remained between 70% and 80%. First, 7 cm × 7 cm × 8 cm plastic flowerpots were filled with nutrient soil and 5–8 plants were sowed in each flowerpot. When the foxtail millet grew to 3–5 leaves, five different concentrations of penoxsulam were applied by indoor pot spraying technology in a similar way to the field experiment. Samples were taken at 5, 10, 15, 20, 25, and 30 days. The spraying machine was 3WP-2000 bioassay spray tower (development of Agricultural Machinery Research Institute of Nanjing Academy of Agricultural Sciences, Nanjing, China). The stepping distance of the spray tower was set to 1340 mm, the traveling speed was 497 mm/s, the spray flow rate was 390 mL/min, and the effective spray range width was 350 mm.

### 4.4. Indicators and Methods of Determination

After 20 days, samples of 0.5 m × 0.5 m (0.25 m^2^) were collected on each piece of soil according to the “5-point sampling” method. The number and weight of four kinds of weeds in each plot were measured, and the control effect of number and fresh weight inhibition rate was calculated by the following formula:

(1) $$The\;control\;effect\;of\;number\left(\%\right)=\frac{Number\;of\;weeds\;in\;control\;area-Number\;of\;weeds\;in\;treatment\;area}{Number\;of\;weeds\;in\;control\;area}$$

(2) $$The\;fresh\;weight\;inhibition\;rate\left(\%\right)=\frac{Fresh\;weight\;of\;weeds\;in\;control\;area-Fresh\;weight\;of\;weeds\;in\;treatment\;area}{Fresh\;weight\;of\;weeds\;in\;control\;area}$$

The stem diameters and above-ground fresh weight of foxtail millet plants were determined with vernier caliper and analytical balance, and then put it in an oven at 105 °C for approximately 30 min, dried below 80 °C to a constant weight, and then the above-ground dry weight was measured. In order to determine the above fresh weight, the above-ground parts of the foxtail millet seedlings were collected and the fresh above-ground weight (g) was determined. For the determination of the above-ground dry mass, the above-ground section was put into the oven, deactivated for 15 min at 105 °C, dried at 80 °C, and weighed.

Then, a 0.1 g sample of foxtail millet leaves was weighed, 1.8 mL of 0.05 mol L^−1^ phosphate buffer with a pH of 7.8 was added, and the mixture was homogenized and frozen. Put the homogenate into a centrifuge and centrifuge at 4 °C with 12,000× *g* for 15 min. The SOD, POD, and CAT activities were determined by extracting the supernatant. The samples were determined by ultraviolet spectrophotometer (Shunyu Henping Instrument, LLC, Shanghai, China) [43].

SOD activity (EC 1.15.1.1) was detected using the nitro blue tetrazolium (NBT) method. The reaction mixture (5 mL) includes phosphate buffer (50 mmol L^−1^) with a pH of 7.8, L-methionine (13 mmol L^−1^), NBT (0.075 mmol L^−1^), EDTA (0.1 mmol L^−1^), riboflavin (0.002 mmol L^−1^), and 20 mL enzyme extract. The reaction mixture was irradiated for 15 min at 25 °C, under the illumination of 4000 lx. One active unit corresponds to the inhibition of 50% NBT in the light environment. The absorbance was measured at 560 nm and the entire reaction mixture without irradiation was used as control.

The POD activity (EC 1.11.1.7) was determined using the guaiacol method; 3 mL of reaction solution was added to 20 µL of enzyme extract. The reaction solution contained 3 mL of 100 mmol L^−1^ phosphate buffer with a pH of 6.0, 19 µL of guaiacol, and 28 µL of 30% H_2_O_2_. The activity of POD was determined by the change in absorbance at 470 nm for 3 min.

The CAT activity (EC 1.11.1.6) was determined using the ultraviolet absorption method. A total of 2.7 mL of Tris-HCl and 50 µL of H_2_O_2_ were added to 20 µL of enzyme extract. After H_2_O_2_ was hydrolyzed, it was continuously determined at 240 nm for 3 min.

MDA was measured using TBA. A total of 0.4 g of fresh foxtail millet leaves were evenly mixed with 5 mL of 0.1% trichloroacetic acid (TCA) and 5ml of 0.5% TBA was added into the mixed solution and mixed well. The reaction mixture was placed in boiling water for 15 min, and then placed in ice water for rapid cooling. Subsequently, the mixed liquid was centrifuged at 4 °C of 3000× *g* for 15 min. Subsequently, the absorbance of the supernatant was measured at 532 nm and 600 nm. This index has been implemented in three technical and biological repeated experiments.

After harvesting, measure the traits with a ruler and a scale: ear length, ear weight, and 1000 grain weight. All of the land was harvested, threshed, and dried. Every area of foxtail millet was measured accurately, which is equivalent to the output of one hectare.

### 4.5. Data Analyses

All the experiments were a completely random design repeated three times. All data were expressed by mean ± standard deviation (SD). Two-way ANOVA (IBM SPSS Statistics for version 27, IBM Corporation, Armonk, NY, USA) was conducted to determine whether the results of the experimental repeats differed significantly. At the same time, between different treatments, Duncan was used to identify statistically significant difference and reached a significance level of *p* < 0.05.

## 5. Conclusions

Among the five spraying dosages, the 1/2X spraying dosage has a good control effect on *A. retroflexus* and *E. crus-galli* but a poor control effect on *C. album* and *D. sanguinalis*, which is safe for foxtail millet, produces little phytotoxicity, and can quickly restore growth. The 1X spraying dosage has a good control effect on *A. retroflexus* and *E. crus-galli* and has a certain control effect on *C. album* and *D. sanguinalis*. However, the effect is insignificant; this group is relatively safe for foxtail millet and has a low degree of phytotoxicity. The control effect of the 2X spraying dose on the four dominant weeds in the grain field is good, and has a significant degree of phytotoxicity on Jingu 21, which leads to a 3.83% reduction in the yield of Jingu 21. The 3X and 4X spraying dosages effectively control the four types of weeds in fields but exhibit stronger phytotoxicity on Jingu 21. The 1/2X and 1X spraying dosages are more effective in controlling gramineous weeds in fields. However, due to the potential harm to crops, using other spraying dosages in the field is not recommended.

## Figures and Tables

**Table 1:** Effect on yield traits and yield.

Variety	Treatments	Ear Length (cm)	Ear Weight (g)	1000 Grain Weight (g)	Yield (kg/ha)
Jingu 21	CK	25.63 ± 1.0 ab	32.98 ± 3.1 a	3.03 ± 0.1 a	4002.00 ± 147.50 b
	1/2X	25.20 ± 1.0 ab	33.78 ± 3.5 a	2.98 ± 0.1 ab	4945.81 ± 230.07 a
	1X	26.60 ± 1.5 a	34.23 ± 3.1 a	2.87 ± 0.1 bc	4855.76 ± 130.24 a
	2X	23.16 ± 0.8 bc	30.47 ± 2.4 ab	2.82 ± 0.1 c	3848.59 ± 139.15 b
	3X	24.15 ± 0.6 bc	27.75 ± 2.8 b	2.8 ± 0.1 c	3771.89 ± 170.18 b
	4X	21.38 ± 0.6 c	22.98 ± 1.7 c	2.67 ± 0.1 d	3101.55 ± 222.00 c

Note: Comparison of treatment at the same day with different concentrations, with lowercase letters showing marked differences (*p* < 0.05).

**Table 2:** Physical and chemical properties of tested soils.

Total N (g/kg)	Total P (g/kg)	Total K (g/kg)	Available K (mg/kg)	Available P (mg/kg)	Alkaline N (mg/kg)	Organic Matter (g/kg)	pH
1.04	1.12	18.75	291.09	23.62	51.84	23.36	8.2

## Data Availability

The data that support this study are available upon reasonable request from the corresponding author.
